# Real-time Ecological Assessment of the Context of mental and physical Health (REACH) on Curious

**DOI:** 10.21203/rs.3.rs-8171682/v1

**Published:** 2025-12-17

**Authors:** Mathilde M. Husky, Larissa Hunt, Andrew Leroux, Debangan Dey, Kevin Conway, Celine Vetter, Vadim Zipunnikov, Rene Choudhari, Mirelle Kass, Michael Leyden, Yuki Kotani, Tarannum Lateef, Giovanni Salum, Ananya Swaminathan, Yao Xiao, Arno Klein, Michael Milham, Kathleen R. Merikangas

**Affiliations:** INSERM U1219, University of Bordeaux; National Institute of Mental Health; University of Colorado Anschutz Medical Campus; National Institute of Mental Health; National Institute of Mental Health; National Institute of Mental Health; Johns Hopkins Bloomberg School of Public Health; National Institute of Mental Health; Child Mind Institute; Child Mind Institute; Child Mind Institute; National Institute of Mental Health; Child Mind Institute; National Institute of Mental Health; Child Mind Institute; Child Mind Institute; Child Mind Institute; National Institute of Mental Health

**Keywords:** Ecological Momentary Assessment, MindLogger, Curious, Applet, Digital mental health, Emotions Behavior, Context, Reactivity, Adults, Children, REACH

## Abstract

Ecological Momentary Assessment (EMA) is widely used in mental health research to capture internal states and experiences as they occur in real-world settings, yet the lack of standardization in EMA content, study design, analytical approaches and data collection platforms has hindered comparability across studies and limited progress in mental health research. We have developed standardized, open-source tools for EMA with the **R**eal-time **E**cological **A**ssessment of the **C**ontext of mental and physical **H**ealth (REACH) at the National Institutes of Health Clinical Center, to characterize dynamic profiles of physical and mental health across the lifespan. The REACH applet is integrated into Curious, a secure, open- source, configurable, and scalable platform through a collaborative effort with the Child Mind Institute. We describe the content, materials, and methods of REACH shared under a Creative Commons license to promote access and harmonization of these tools in global settings across the life span.

## Introduction

Experience sampling ^1^ or Ecological Momentary Assessment (EMA) ^2^, is increasingly used to characterize various mental and physical health conditions as they unfold in daily life. EMA relies on real time self-reports of context, mood, thoughts, stressors, pain, and behaviors reported by the individual in their natural environment, assessed at semi-regular intervals. The repeated nature of the assessments allows for the study of both within- and between-person dynamic processes related to psychological states, behavior and health outcomes ^3-9^. Additionally, EMA enables researchers to identify proximal predictors of specific outcomes such as negative affect ^10^, suicidal ideation ^11-15^, substance use ^16,17^, and physical health events such as migraine ^18^. There are also growing efforts to incorporate EMA in intervention studies (e.g., Ecological Momentary Interventions, EMI) to enhance personalized medicine approaches in both adults ^19,20^ and children ^21^ through the derivation of fine-grained individual profiles to identify personalized treatment targets ^22^, monitor patients undergoing interventions ^23-25^, and deliver just-in-time adaptive interventions (JITAIs) ^26,27^.

EMA has a critical role in accessing inherently subjective experiences which require self-reports of internal psychological or physical states such as affect, feelings, energy, thoughts, worries, or pain. Self-reports also play a central role in characterizing daily life experiences and stressors which may inflence moment-to moment affect ^7^. A considerable body of research has underscored the utility of EMA to access internal states and experiences in both adults ^3,13,28,29^ and youth ^21,30,31^, states that cannot be inferred from indirect measures. EMA has had particular utility for collecting self-reported experiences of craving and the physical and social context of addiction to identify personalized proximal predictors of subsequent use to inform prevention efforts ^32^. .Despite substantial efforts to infer emotional states such as depressed or sad mood through facial expressions ^33^ and speech patterns ^34^, such indirect measures remain indices of internal states and may not reflct an individual’s interpretation of their own emotional and physical states and well-being ^35^.

Most of the progress in EMA has emerged through applications to individual studies rather than larger scale efforts with standardized content and procedures. This lack of standardization has led to widespread heterogeneity in the content, study design and administrative procedures that impede aggregation of EMA research, even on the same topic. Ongoing efforts to centralize the questions, format, and procedures for EMA will begin to address this issue ^36-39^. There are also multiple collaborative studies that are establishing procedures to expand ancillary measures in EMA studies to incorporate passive monitoring with wearable devices. While combining EMA with passive measures such as physical activity, sleep, exposure to light and sound, heart rate, prosody, and ambient temperature, can provide a comprehensive profile of multiple domains in real time, the data management and analytic challenges are daunting.

As EMA research has shifted from paper-pencil, minicomputer and research administered devices to participant smartphone as the primary platform for administration, there has also been substantial variation in the technology component of EMA. Numerous administrative platforms, in-house device-specific programs, or apps have been created both commercially or otherwise, all with inherent limitations and lack of scalability ^40^. The development of a flexible data collection platform for EMA can facilitate data visualization and centralize wearable data that track other health domains such as accelerometry, heart rate, cognitive testing and collection of biologic samples. To address these challenges, the *MindLogger* platform was conceived and prototyped by the MATTER Lab within the Child Mind Institute (CMI) in New York. The *Mindlogger* platform has been updated and reprocessed in a newly introduced platform entitled Curious. This non-commercial open-source, secure, configurable and scalable platform was designed to support clinical measures, mobile assessments such as EMA, real time cognitive tasks, and interventions on a common platform without requiring study team programming expertise.

This paper describes the culmination of two decades of collaborative empirical research to expand and harmonize the content and procedures for collecting EMA with the **R**eal-time **E**cological **A**ssessment of the **C**ontext of mental and physical **H**ealth (REACH) applet on *Curious*. As a central measure of temporal dynamics of health, behaviors, and emotions in the Rhythms and Blues Study at the NIH Clinical Center ^41^, REACH characterizes the profiles of mental and physical health and the dynamics of a comprehensive range of homeostatic systems and contextual factors in daily life. REACH is an expanded version of comprehensive EMA modules that were developed through the mobile Motor Activity Research Consortium for Health (mMARCH), a multisite collaborative network designed to develop common procedures and analytic methods for accelerometry in studies of mood disorders. Standard EMA modules on mood, context, and life events were expanded to enhance the reliability of domains extracted from wearable accelerometry devices (e.g., physical activity, sleep and circadian rhythmicity) with modules on sleep patterns and quality ^42,43^ and exercise and physical activity ^44^. This combined passive and active monitoring has enabled us to investigate the real time bidirectional dynamics of mood states, activity, and sleep patterns ^45^. Here we describe the platform, versions, contents, and procedures that can be accessed through a Creative Commons license.

## Methods

### The CuriousPlatform

The Curiousplatform is a secure, scalable, and user-friendly data collection platform for mental health research ^46^. Its functionality has been extensively described elsewhere ^40^. It seamlessly operates on both IOS and Android systems and is accessible anywhere in the world that allows uncensored access to the Internet. Curiousenables the administration of EMA alongside additional assessments such as cognitive tasks. In addition, it meets all key requirements for an EMA platform regarding multiple response format options, data management, and security. Here, we focus on how REACH works on Curious. Briefly, research staff download the Curiousdesktop application from the website and install the dashboard. The staff-facing dashboard enables researchers to control all study parameters, monitor participation in real time, and access pooled participant data. Researchers can schedule EMA notifications and set a limited time window for responses to beconsidered valid (e.g., 60 minutes after each notification). On participants’ end, they are asked to download the Curiousmobile application from Google Play or the App store on their personal smartphone to use throughout the study as an electronic diary. The Curiousapp will send notifications several times a day and prompt participants to answer each EMA survey.

#### Data storage

Curiousensures that participants receive all EMA notifications and can complete EMA assessments even when their Internet access is intermittent. Data are stored locally, on the person’s own phone and the app automatically uploads the data once the Internet connection allows it. This functionality requires participants to have a minimum of 1.5 MB of free storage space on their phone.

##### Data confidentiality and safety

Responses to EMA assessments are not stored with participant personal information. Both sets of data are stored and encrypted separately. Curioushas HIPAA-compliant agreements with MongoDB Atlas, AWS, and Google. Curiousemploys robust data security measures, including SSL/TLS encryption for secure transmission and AES 256-bit encryption for data storage. User information, such as emails and names, is encrypted within the database. Text based response data are end-to-end encrypted using a key that is specific to the applet, remaining secure and inaccessible by the Curious team throughout transmission and storage. Researchers can choose to store data on CMI cloud servers or their own infrastructure, with data remaining encrypted even if the database is compromised. Detailed security documentation is available on the Curious website ^46^.

#### Versions and Content

##### Four versions of REACH

**Figure 1:**
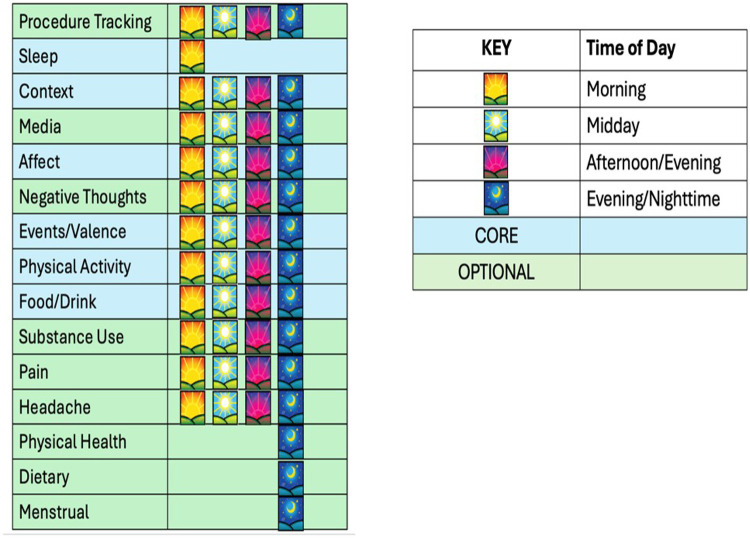
Core and optional modules of REACH by time of day.

We have developed four versions of REACH: a full adult version (FAV), a short adult version (SAV), a full child version (FCV), and a short child version (SCV). The adult versions were designed for use among persons 12 years old and older and the child versions were adapted for language, response options, and images suitable for children ages 6 through 11. Shortened versions that include the key items from each of the modules are available for both adults and youth. Figure 1 presents the core and optional modules of the REACH in the FAV by the time of day of their administration. Standard variable names, and question/response format are used across all four versions to facilitate data management and analysis of different versions of the REACH within a study. Below, we briefly describe the content of each of the modules assessed in the FAV, as it is the version with the most variables.

###### 1. Procedure tracking

Procedure tracking modules are used to monitor the use of other wearables and data collection methods that include accelerometry, light trackers, heart rate monitors, and biological sample collection in the NIMH Rhythms and Blues Study. Participants are prompted to report wear time and removal of ancillary devices. In addition, Curiouscan provide notification reminders to participants for timed ancillary measures ^40^.

###### 2. Sleep

**Figure 2:**
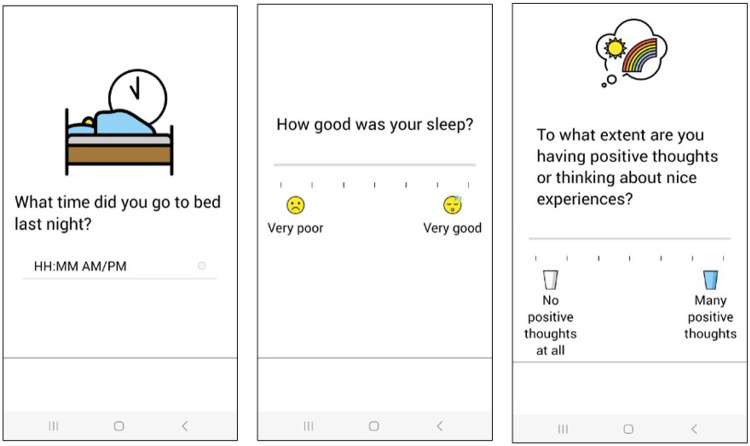
Sample Screenshots of REACH Items from the Sleep Module (left and center) and the Thoughts Module (right).

Sleep is assessed once per day, in the morning. specific items have been designed to collect the information for sleep diaries that have been recommended for deriving features of sleep from accelerometry by the American Sleep Society ^47,48^. In the short version, respondents are asked to indicate the sleep duration on the previous night (in 30-minute increments), bedtime, wake time, and self-reported sleep quality. The long version includes reports of lights off time, removal of devices, wake time, and the number of overnight awakenings. In addition, information on specific sleep problems and medication/sleep aids is also collected. Figure 2 displays two items from the sleep module.

###### 3. Context

At each assessment, respondents are invited to report their location using Curious’sGPS locator. Clicking ‘Get location’ when prompted will collect the participant’s GPS location. However, the participant can choose to skip this question, should they not want their coordinates to be known. This question is the only question within REACH that has a built-in skip feature. Respondents then report where they are, who they are with, and what they are doing using multiple choice options. The long version further asks participants to report the same information during the time since their prior assessment. Participants are presented with multiple response options covering typical daily life contexts. This section allows classification of both the physical and social contexts that may influence emotions and behavior ^49^, as well as exposure to outdoor vs indoor settings that can be used to assess light and temperature exposure ^50,51^.

###### 4. Social Media

The Social Media module was included in the latest version of the application due to the increasing shift to online social communication and information acquisition, as well as the link between social media use and mental health ^52^. The aim is to quantify time, type, and interactive nature of online activity, including video game and social media use. Respondents are further asked to indicate their level of engagement in specific activities on social media (e.g., posting comments, pictures, videos), and the valence of their posts/comments and perceived reactions from others.

###### 5. Affect

Affective states are assessed using four dimensions of the mood circumplex model of affect, including: happy/sad, calm/anxious, inactive/active, and tired/energetic on a 1 to 7 Likert scale ^53^. Additional items that characterize mood disorders have been added to the long version include: concentration or focus, irritability, quickness of thinking, fidgetiness and level of pleasure/enjoyment (e.g., anhedonia) on the same 1 to 7 scale. Fluctuations in symptoms beyond the mood circumplex have been central to defining subgroups of mood disorders ^54^.

###### 6. Thoughts

As a follow up to the affect section, respondents are asked to rate the extent to which they are experiencing positive and negative thoughts (see Figure 2). Endorsement of negative thoughts leads to inquiry about their content and clinical significance. The latter item is used to prompt an optional module assessing active and passive suicidal ideation. As most EMA studies do not assess suicidal ideation, we chose not to include these items in the final protocol shared here. Curioushas the capability of sending alerts to research staff in real time if certain items are endorsed. This feature is critical for clinical oversight and securing approval from ethical boards in some studies investigating suicidal ideation and behaviors.

###### 7. Events

Daily hassles and minor stressors are key contextual variables for understanding a person’s daily life experiences. We adapted prior EMA versions ^5^ that were themselves adapted from event-type checklists ^55^. The assessment is based on identifying the most significant event since the previous assessment, prompting the respondent to type or use voice-to-text to provide a brief verbatim description of details of the event. Respondents then rate the valence, category, social nature, and stress level of each event. Categories include social interactions, getting things done, health-related events, or news-related positive or negative events.

###### 8. Physical activity

Respondents are asked about the level and timing of sedentary, light, moderate and vigorous activity since the last assessment. In the long version, if sedentary activity was endorsed, respondents are asked to specify whether they had been napping, resting, or sitting, and for how long, and whether they fell asleep during that nap and for how long. Participants are instructed about the response options in advance to minimize deliberations in response during the study. The application of EMA to track physical activity and sleep provides more comprehensive characterization of the patterns of these behaviors that can inform intervention studies ^44^. This information has been particularly helpful in scoring activity and sleep assessed by wearable sensors.

###### 9. Food and Drink

**Figure 3:**
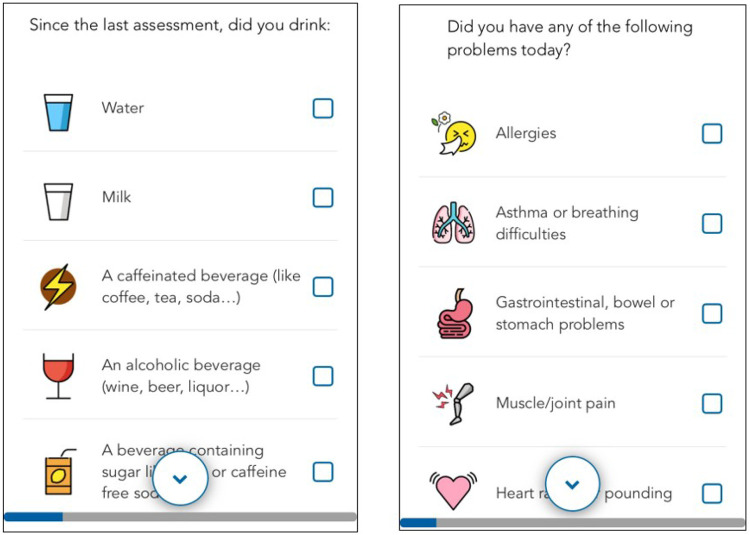
Sample Screenshots of REACH Items from the Food and Drink Module (left) and the Physical Health Module (right). All icons were either freely available or were designed specifically for the study.

The Food and Drink section includes questions on thirst, types and quantities of beverages consumed. Endorsement of alcohol use is followed by questions regarding the type and amount consumed, the time since their last drink, and level of intoxication. Respondents are also asked about how hungry they currently feel, how many times they have eaten since the last assessment, how much they had to eat, and when they last ate. Food groups are assessed at the end of the day as part of the diet module described below. Figure 3 (left side) displays one item from the food and drink module. This combined approach to the collection of dietary information provides a more accurate characterization of diet and nutrition ^56^.

###### 10. Substance Use

The Substance Use Module is part of the full adult version and is not included in the short versions of the REACH. The REACH module has been updated to collect contemporary substance use patterns. The substance use module starts with the assessment of thoughts of using tobacco (including cigarettes and e-cigarettes), cannabis (including flower, vaping, edibles, and other consumables), other drugs (including painkillers, heroin, and fentanyl) and alcohol. Use and timing of actual substances are then assessed. Lastly, respondents who endorsed use of cannabis or other drugs are asked questions about their subjective reaction and social context of use.

###### 11. Pain

The Pain section includes a measure of current pain intensity, physical location (e.g., head, musculoskeletal, gastrointestinal) since the last assessment. This module can be tailored to studies that focus on specific pain conditions, as well as broader impact of non-specific pain on daily life functioning ^57^. Additional items relevant to pain studies may be added on a study-specific basis (e.g., pain interference, catastrophizing, etc.).

###### 12. Headache

The Headache Module is included when a headache has been endorsed in the pain section. This module includes the key features of headaches that are included in traditional headache diaries ^58^. Features assessed include the location, nature of pain, intensity, accompanying symptoms, including visual and neurologic symptoms (e.g. difficulty speaking or thinking, numbness or tingling), and preventative/abortive measures (e.g., medication, rest) taken for headache management.

Triggers, prodrome, and associated symptoms such as sensitivity to light, heat, noise, smell, or physical activity are also assessed. These reported symptoms can be used to provide indices of headache subtypes according to the current diagnostic nomenclature for headache syndromes by the International Classification of Headache Disorders, 3^rd^ edition, (2018). More details of the applications of the headache modules are described elsewhere ^18^.

###### 13. Physical health

At the end of the day, respondents are asked about their overall health for that day. Respondents are asked to identify any health problem they may have experienced and how much these problems bothered them that day. A predefined list of problems is displayed and includes the following: allergies, asthma or breathing difficulties, gastro-intestinal problems, muscle or joint pain, heart racing, feeling faint or dizzy, and feeling lethargic. In the long version, respondents are asked to indicate use of either over the counter or prescription medications from a precoded medication list. This module can be expanded to track compliance with medications in the context of treatment studies. Figure 3 (right side) displays one item from the physical health module.

###### 14. Diet

The Diet Module was developed to facilitate the collection of the timing and size of meals and snacks, as well as the characterization of food groups across the day. Details of respondents’ daily diet are collected at the end of the day due to research showing the reporting of actual caloric intake or estimation by food photography have been shown to be overly burdensome and unreliable ^59^. In the full adult version, at the end of each day, respondents are asked to identify the foods they had during that day within each of five broad categories: (1) meat, poultry or fish; (2) dairy or eggs; (3) fruits or vegetables: (4) snack foods, desserts; (5) bread, grains, or cereals. Within each category, respondents then specify the food groups based on dietary classification of the NIH Daily Food List (https://www.nhlbi.nih.gov/health/educational/lose_wt/eat/diary.htm).

###### 15. Menstrual Cycle

At the end of each day, females are asked to indicate whether they are having their menstrual period. This section can be expanded to collect more details of signs and symptoms and ancillary hormonal measures for studies of reproductive life transitions ^60^.

#### Administration

##### Proposed scheduling patterns

EMA studies have used various time frames and scheduling patterns ^61^. REACH was designed for use 4 times per day over a period of 14 consecutive days to cover both weekdays and weekends in order assess within-person dynamics. Using EMA with these parameters has been shown to be feasible and acceptable in various samples ^38^. Covering both weekdays and weekends is important as there are known differential patterns of affect, contexts, and activities ^62^.

Scheduling the times at which the notifications are sent is set with each study participant individually. The constraints include having the first assessment as close to wake time as possible, and the last assessment as close to sleep time as possible, with the two midday assessments required to be at least 2 hours apart from any other assessment. The timing of collection of biological measures (i.e., salivary cortisol, melatonin) during EMA may vary according to the study goals. For example, awakening cortisol is a standard measure of stress ^63^. Likewise, sleep research relies on accurate reports of bedtime and lights off.

The scheduling pattern used by REACH is fixed, as random sampling has been shown to yield lower compliance ^38,62,64^. notifications occur at the same time every day throughout the full 14 days, except for the weekends or days off school or work where respondents may choose a different set of times based on changes in their normal wake time. For example, one person’s weekday schedule could be: 7:15 AM, 11:30 AM, 4:30 PM, and 9:15 PM and on the weekend, that person’s schedule could be 9:30 AM, 12:30 PM, 4:15 PM, and 10:00 PM.

##### Training staff and participants to use REACH

One of the chief sources of variability and non-compliance in EMA is a lack of systematic training and monitoring of both the staff and participants ^38,62^. It is particularly important to engage participants in the goals and concepts of EMA and receive direct training and piloting of the applet prior to initiation of the EMA protocol. A participant training manual is provided for each of the four versions of REACH and should be shared with the staff who will be administering EMA. These training manuals are available on the MINDS website (url forthcoming upon acceptance of the manuscript for publication).

### Quality Control of Data and Compliance with REACH

#### Data processing and output

Data output is accessible on the staff’s dashboard throughout the study and populates in real time as data are uploaded by participants’ apps. This key feature allows researchers to monitor compliance during data collection and to examine preliminary data during study enrollment.

#### Visualization

The full data dictionary is available on our website along with the other REACH materials, under the same licensing terms. A browser-based dashboard for exploring EMA study data is also available on our website. The tool enables data quality control, compliance monitoring, and exploratory visualization of both trait and time-series variables at subject and study levels. Built with DuckDB, SvelteKit, and Observable Plot, it runs entirely client-side, allowing researchers to analyze sensitive study data without uploading it to external servers. The application provides an efficient workflow for initial data inspection, tracking participant adherence, and generating interactive visualizations to identify patterns and potential issues in the collected data.

#### Data quality control

Quality control comprises two elements: careless responding and compliance. While some level of careless responding can be expected ^37^, further work is necessary to identify and address such patterns. Although the fixed order of questions and responses without a skip option has the advantage of reducing cognitive burden, it also tempts participants to endorse the first set of response options. Systematic identification of dubious responses through inspection of distributions of response time, the joint distribution of survey items, and their individual variability in these quantities is recommended. Surveys that are completed faster than, for example, 95% of “valid” surveys, or surveys where participants respond more than two standard deviations faster than their average time might be flagged for review. More work is needed to investigate the psychometric properties of some EMA variables, including content validity and external validity against other assessments ^65^, variable order, detection of careless responding, and patterns of missing data, and properties of brief versions for each module of the REACH.

#### Monitoring missing data and compliance

Using EMA requires a careful consideration of participants’ compliance with study procedures ^64,66^. As noted above, the applet was programmed in a manner that does not allow participants to skip individual questions within each assessment. This feature prevents within-assessment missing data. Furthermore, if a participant initiates an assessment but is interrupted, Curiousallows for partial EMA surveys to be uploaded when the 60-minute time window ends. It is therefore possible to have truncated assessments (responses to the first questions only), missed assessments (no responses), and fully completed assessments (all questions answered). Compliance can be defined as the proportion of fully completed assessments over all possible assessments. A less stringent definition would be to consider all assessments that were started with a completion of 50% or more of all question sets or modules within each assessment. Although branching and skip patterns generate variability in the number of questions within each assessment, output from Curiousallows identification of the modules asked midway through the full assessment. Any response after this point for incomplete assessment would be considered valid and serve to determine compliance. This is a central parameter that should be monitored in ensuring the feasibility of a new application of REACH. Incoming data should be monitored continuously for completeness, both to assess potential technical issues, and to evaluate whether the current study protocol results in acceptable and/or expected missingness.

Curiousallows researchers to document all EMA notifications, the time between a notification and a response, the time lapse between the response to the first item of an assessment and the response to the last item. These parameters allow for a more detailed assessment of participant engagement. Our team is currently working on designing a shiny app designed to monitor participants’ compliance in real time, and scripts to analyze compliance in various ways. This information will be made available on our website.

#### Assessment duration

Estimates of assessment duration are based upon pilot data collected within NIMH’s Rhythms & Blues Study. Briefly, the Rhythms & Blues Study is a longitudinal family study of motor activity, circadian rhythms, and mood states in adults and children with and without mood disorders. The study received the approval of the National Institutes of Health (NIH) Institutional Review Board (IRB), and all participants provided written consent. Based upon pilot data collected from N=81 adults, the median duration of the full adult EMA is 7.0 minutes and a mean of 8.6 minutes (SD = 6.4). Pilot data collection on the Short Adult Version and on both child versions is underway.

#### Enhancing participation and compliance

Participating in EMA can be perceived as burdensome to participants, especially with longer EMA assessments, or if combined with wearables or other repeated measures. EMA survey length has been associated with lower compliance (data quantity) and careless responding (data quality) in a sample of young adults ^37^. To minimize such biases and enhance compliance ^66^, we recommend offering incentives commensurate with compliance when compatible with local IRB requirements. In preliminary data collected in the context of NIMH’s Rhythms & Blues Study, 80.0% of EMA assessments were initiated by participants, and 76.5% were initiated and completed in full.

#### Statistical tools for analyses of Ecological data

EMA data record multiple within-day self-reported assessments of participants’ behavior and well-being on different mixed-type scales, e.g. affective states (ordinal scale), the occurrence of daily life events, headaches, diet module (binary scale), and pain reporting (truncated scale). Analyzing such data presents statistical challenges due to its intensive longitudinal nature, complex patterns of missingness, the difference in reporting scales, within- and between-subject variability, and integrating multimodal data streams ^67^. We have developed a data analysis pipeline that standardizes quality control, data processing, handling of missing data, and generates secondary variables of interest in a reproducible manner.

Approaches to the analysis of complex multilevel multidomain intensively repeated data extracted from EMA are particularly challenging ^68,69^. In addition to group averages, characterization of within subject patterns and variability both within and between domains is central to addressing the range of questions that can be assessed by EMA. To address the difference in reporting scales across the measures, our research team developed Functional Principal Component Analysis (FPCA) methods for mixed data types, allowing for the effective identification of differences in the diurnal dynamics of affective states across mood disorders by reducing the dimensionality of the data and reporting principal modes of variability ^18,70^. In addition to characterization of within- and across-day dynamics using approaches such as fragmentation metrics has been used to measure behavioral instability, including mood and attention shifts ^71^. We have introduced multimodal integrative dimension reduction techniques such as Joint and Individual Variance Explained (JIVE) ^72^ and Similarity-Based Multimodal Regression ^73^ to explore associations between clinical outcomes and multimodal EMA data. Inter-and intra-domain correlations between affective states across time are estimated with Generalized Linear Mixed Models, Generalized Estimating Equations (GEE) ^45^ and Dynamic Structural Equation Modeling (DSEM) ^28^ to examine whether the correlations differ by study subgroups or demographic correlates. We have also developed dynamic prediction models that distinguish within- and between-subject effects as predictors of short-term adverse behavioral health outcomes, such as headaches ^18^. These statistical methods enhance interpretability and enable translational discoveries for clinical applications.

### Accessing REACH on Curious

REACH Curiousapplets and related training materials are shared under an Attribution- Non-Commercial-Share Alike 4.0 International Creative Commons license agreement (CC BY-NC-SA 4.0). Accessing the instrument and training manuals requires registering on our website and accepting the terms of the licensing agreement. specifically, the CC BY-NC-SA 4.0 license requires that reusers give credit to the creator by including the following language:

« This work was supported by the Intramural Research Program of the National Institute of Mental Health (ZIA MH002804 and ZIA MH002954, PI: Dr Kathleen R. Merikangas). The views and opinions expressed in these materials are those of their authors and should not be construed to represent the views of any of the sponsoring organizations, agencies, or U.S. Government ». The licensing terms further allow reusers to distribute, remix, adapt, and build upon the material in any medium or format, for noncommercial purposes only. If others modify or adapt the material in any way, they must license the modified material under identical terms.

All REACH materials including the applet and training manuals are currently available in English. In addition, translations into Korean, Portuguese, Spanish, French, and Urdu are in progress and will be made available soon. Any group requiring additional languages for the REACH materials should consider the steps listed on our website for translation and adaptation of the instrument and related materials.

## Discussion

### Summary

This paper describes the culmination of multisite efforts to expand and harmonize the content and procedures for EMA by developing standardized, open-source tools for data collection with the REACH app. This app has been fully integrated into Curious, a secure, open- source, configurable, and scalable platform through a collaborative effort with the Child Mind Institute in New York. As the central measure of temporal dynamics of health, behaviors and emotions in the Rhythms and Blues Study at the Clinical Center, REACH is combined with other wearables to characterize the dynamics of emotional states and their interrelationships with a comprehensive range of homeostatic systems and contextual factors in daily life.

### Challenges and Ongoing Developments

Our efforts to standardize the content, procedures and analyses of REACH described herein begin to address the challenges in interpretation of aggregate EMA research on mental health that is now impeded by widespread variation in samples, content, procedures, and analytic methods. The goal is to enhance numerous ongoing efforts to develop and share common

survey items and procedures and analytic methods ^39^ for diverse EMA applications designed to address the following ongoing challenges in EMA research.

First, the cross-sectional nature of EMA over brief time periods of 1–2 weeks in descriptive studies limits the generalizability of the profiles both at the group and individual level. An increasing number of EMA studies use a “burst sampling” approach that involves brief periods of EMA to cover a year or more, often with abbreviated modules to minimize time for completion ^69,74,75^. Information on the longer-term stability of EMA will be available from our current protocol that includes systematic follow up of EMA several times a year. The repeated use of EMA over longer time periods will facilitate our understanding of within subject variation that is central to our understanding of naturalistic course, treatment response and seasonal variations ^76,77^.

Second, despite evidence for the feasibility and acceptability of EMA, it remains burdensome, particularly over longer periods of administration. Dissipation in compliance is universal in EMA studies, particularly those studies that involve longer time periods of assessment of clinical samples ^78^. Additional work is needed to test the validity of shorter versions, and to identify procedures and incentives for EMA across multiple study designs and contexts that will minimize burden and maximize compliance.

Third, a major challenge in the *mMARCH* research network concerns the development of REACH versions and data integration across diverse study aims, samples and age groups. Different versions of the REACH with varying core and optional models for different age groups and informants will facilitate the seamless integration of versions tailored to specific populations and their comparability across studies.

Fourth, our experience in harmonizing accelerometry across multiple sites in the mMARCH consortium has highlighted not only widespread variability of content and procedures for accelerometry measures, but also the lack of uniform diagnostic methods, symptom measures, sample descriptions, collection of covariates and characterization of clinical status at the time of the research (e.g., state vs trait) that has also impeded aggregation of the results ^79^. Therefore, in addition to standardization of accelerometry and EMA procedures, we have also expanded efforts to employ common diagnostic, clinical, health assessments and current state measures to enhance the comparability and generalizability of this research. Development of an infrastructure to facilitate the process of establishing an EMA data repository that builds upon on our multisite accelerometry data base from the mMARCH network is now underway.

**Figure 4:**
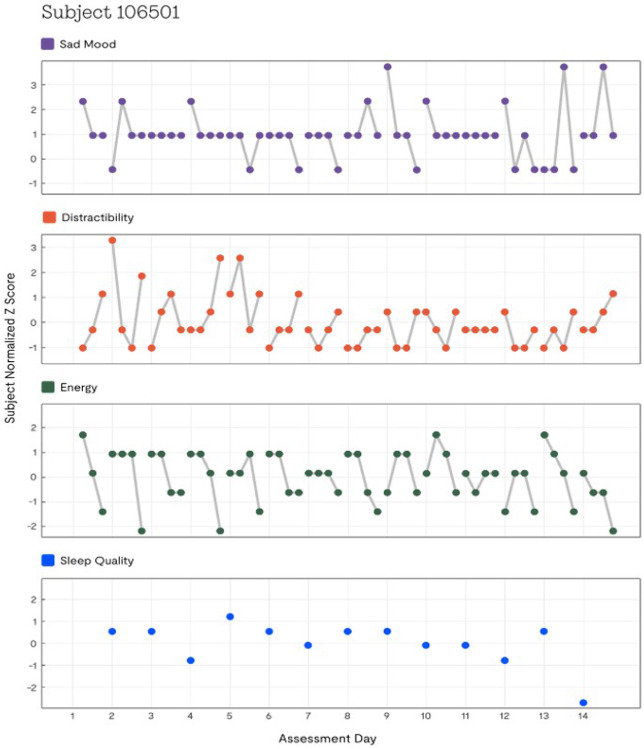
Individualized REACH profile

Fifth, the shift from the traditional descriptive characterization of EMA of limited benefit to participants to potential interventions based on individualized EMA will require embedding EMA in a longer term, controlled research framework. There are growing efforts to develop tools for visualization of EMA that can be used to provide individualized feedback that is likely to enhance compliance with EMA, particularly over longer time periods than those used in current research application ^80,81^. Derivation of individualized profiles across multiple domains can be used to inform both individuals and clinicians about within and between domain fluctuations. For example, Fig. 4 depicts an example of a 14-day profile of averages of mood, distractibility, energy, and sleep in an individual study participant in our study. Using fragmentation metrics, we previously illustrated the utility of such profiles for interventions in people with Bipolar I Disorder based on our finding that instability of energy and attention were more important treatment targets than mood ^28,71^, suggesting that interventions for mood stabilization in this condition may be misplaced ^82^.

## Conclusions

Collaborative efforts are now underway to build potential interventions for the domains that have emerged as sources of distress in our EMA research including mood, sleep, social anxiety ^83^, internet use and substance use. Although we are extending profile feedback and potential interventions to non-clinical samples, there is limited information on the effectiveness of programs to stabilize some of the key domains in people without clinically significant disorders who could nevertheless benefit from personalized approaches with guided feedback. Parallel efforts are also underway to pilot longer term, parent versions, feedback profiles and open-ended participant-administered prompts using the *Mirror* applet. With clinical experts and AI technology, we are developing vetted educational materials and online interventions, and clinical flags that can alert study personnel regarding need for interventions. Finally, along with other wearables and physiological sampling, a toolkit of cognitive tasks entitled Merit has been translated to the Curious platform. These tasks can be combined with domains assessed on the REACH app to provide a more comprehensive multi-domain profile of the dynamics of these systems in real time.

## Data Availability

There is no data presented in this manuscript, with the exception of pilot data from the ongoing Rhythms and Blues study. Because the study is ongoing, the data cannot be made available at this time.

## Code availability:

Scripts for data analysis of REACH variables will be made available on our website upon acceptance of the manuscript.
